# Reaching Out from Europe to the Globe: The International Journal of Psychiatric Trainees

**DOI:** 10.1192/j.eurpsy.2024.389

**Published:** 2024-08-27

**Authors:** F. Santos Martins, D. Cavaleri, D. Zani, L. Tomašić, A. Kibitov, J. King, K. Markin, J. Sprengens, H. Ryland, L. Stirland, A. Seker

**Affiliations:** ^1^Department of Psychiatry, Centro Hospitalar Universitário S. João (CHUSJ); ^2^CINTESIS; ^3^Neurosciences and Mental Health Department, Faculdade de Medicina da Universidade do Porto (FMUP), Porto, Portugal; ^4^Department of Medicine and Surgery, University of Milano-Bicocca , Monza, Italy; ^5^Psychiatry, Psychiatric Services Grisons, Chur, Switzerland; ^6^University Psychiatric Hospital Vrapče, Zagreb, Croatia; ^7^-, -, Russian Federation; ^8^Psychiatry Division, Imperial College London, London, United Kingdom; ^9^Kirov Military Medical Academy, -, Russian Federation; ^10^UMC Utrecht Brain Centre, UMC Utrecht, Utrecht, Netherlands; ^11^ Oxford Health NHS Foundation Trust; ^12^ NIHR Oxford Health Biomedical Research Centre; ^13^Department of Psychiatry, University of Oxford, Oxford; ^14^University of Edinburgh, Edinburgh; ^15^ Institute of Psychiatry, Psychology & Neuroscience, King’s College London; ^16^South London and Maudsley NHS Foundation Trust, London, United Kingdom

## Abstract

**Introduction:**

The *European Journal of Psychiatric Trainees* was founded in 2022 as the official journal of the European Federation of Psychiatric Trainees (EFPT) to offer a peer-reviewed open-access scientific journal with minimal article processing charges. The journal is edited by trainees and early career psychiatrists and published its first issue in July 2023. The journal aims to facilitate publishing experience and opportunities for trainees. To reflect the global identity and inclusivity of psychiatric research, the journal changed its name in 2023 to become the *International Journal of Psychiatric Trainees.*

**Objectives:**

To present the *International Journal of Psychiatric Trainees*, the successor of the *European Journal of Psychiatric Trainees*, and other practical aspects related to the article submission.

**Methods:**

We will reflect on the *International Journal of Psychiatric Trainees*, focusing on what this name change will imply for the journal’s scope, mission and readership.

**Results:**

Due to training programmes’ requirements or out of interest, psychiatric trainees are encouraged to conduct scientific research. However, several known barriers to scientific publishing exist, ranging from a lack of mentorship and supervision to limited scientific support. Like the *European Journal of Psychiatric Trainees*, the *International Journal of Psychiatric Trainees* continues to be an open-access, double-blind peer-reviewed journal with minimal/no publication fees that publishes original and innovative research as well as clinical, theory, perspective, and policy articles and reviews in the field of psychiatric training, psychiatry, and mental health.

Since the difficulties and needs in creating research output are not exclusive to European trainees, the journal will become more attractive to readers and authors from other countries while increasing the diversity of articles.

The first *International Journal of Psychiatric Trainees* issue will be dedicated to the 31st EFPT Forum with the theme “Trainee Mental Health”, containing articles reporting on the projects from National Psychiatric Trainee Associations looking into trainee mental health. Submissions for the regular edition remain open, and articles should be submitted through the manuscript submission platform (https://ijpt.scholasticahq.com)

**Conclusions:**

The *International Journal of Psychiatric Trainees* aims to be an educative scientific journal for psychiatric trainees and other psychiatry and mental health researchers. The name change and its increased openness will help the authors reach a wider readership while the journal can feature a more comprehensive record of psychiatric research through its global scope.

**Disclosure of Interest:**

None Declared

